# Research on multidisciplinary dynamic nursing strategies for incontinence-associated dermatitis in ICU patients based on predictive nursing

**DOI:** 10.1097/MD.0000000000048619

**Published:** 2026-08-14

**Authors:** Shuwei Huang, Xuebin Hao, Yang An, Yimiao Wang, Jiantong Liu

**Affiliations:** a Department of Critical Care Medicine, The First Affiliated Hospital of Hebei North University, Zhangjiakou City, Hebei, 075000, China.

**Keywords:** incontinence-associated dermatitis, intensive care unit, multidisciplinary collaboration, nursing quality, predictive nursing

## Abstract

This study aims to explore the impact of a multidisciplinary dynamic nursing strategy constructed based on the concept of predictive nursing on the incidence, severity, healing status and nursing quality of incontinence-associated dermatitis (IAD) in Intensive Care Unit (ICU) patients and provide a scientific basis for the clinical prevention and control of IAD. A retrospective cohort study design was adopted, selecting 300 patients admitted to the ICU of a tertiary Grade A hospital between January 2023 and December 2024. Based on the time point when the hospital implemented the “Multidisciplinary Dynamic Nursing Strategy Based on Predictive Nursing” starting January 2024, patients were divided into a control group (n = 132, receiving routine care before implementation) and an intervention group (n = 168, receiving multidisciplinary dynamic care after implementation). The control group followed the routine IAD prevention protocol; the intervention group additionally implemented predictive risk assessment, multidisciplinary collaborative intervention, informational nursing support, and quality monitoring. The incidence, severity, healing status of IAD, ICU length of stay, nursing compliance, and quality indicators were compared between the 2 groups. There was no statistically significant difference in the general data between the 2 groups (*P* > .05), indicating comparability. The incidence of IAD in the intervention group was significantly lower than that in the control group (14.3% vs 28.8%, χ2 = 8.45, *P* = .004); the severity of IAD was milder in the intervention group (Mild: 75.0% vs 57.9%, *P* = .022). The complete healing rate of IAD was significantly higher in the intervention group (91.7% vs 65.8%, *P* = .016), the healing time was significantly shorter (7.2 ± 2.9 days vs 9.7 ± 3.6 days, *P* < .001), and the ICU length of stay was shorter (13.1 ± 4.9 days vs 15.4 ± 5.8 days, *P* = .043). Nursing compliance and quality indicators in the intervention group were superior to those in the control group, including daily risk assessment compliance rate, timely warning response rate, nursing record completeness rate, and operational consistency (all *P* < .01). The multidisciplinary dynamic nursing strategy based on the concept of predictive nursing can effectively reduce the incidence and severity of IAD in ICU patients, promote skin healing, shorten the length of stay, and significantly improve nursing compliance and quality.

## 1. Introduction

Incontinence-associated dermatitis (IAD) is an inflammatory skin lesion caused by prolonged contact of the skin with urine, feces, or other moisture-associated excreta. It is one of the significant types of moisture-associated skin damage. The pathogenesis of IAD primarily involves enzymes in urine and feces damaging the stratum corneum, leading to impaired skin barrier function and subsequently triggering an inflammatory response.^[1–3]^ IAD mainly manifests as erythema, erosion, exudation, and even secondary infections in the perineum, buttocks, and inner thighs, severely affecting patient comfort and quality of life, prolonging hospital stays, and increasing healthcare costs. Studies have shown that Intensive care unit (ICU) patients, due to critical illness, limited mobility, high incidence of incontinence, and weakened skin barrier function, are a high-risk population for IAD, with incidence rates reaching 20% to 40% or even higher.^[4–8]^

Currently, numerous studies worldwide have confirmed that routine incontinence care can reduce the incidence of IAD to some extent. However, several challenges persist in clinical practice. The nursing model often relies on “intervention after problem occurrence,” lacking early risk identification and dynamic monitoring. The nursing process is often dependent on individual experience, making it difficult to establish systematic and continuous prevention and control mechanisms.^[9–11]^ Furthermore, the occurrence of IAD is closely related to multiple factors such as the patient’s nutritional status, medication use, infection control, and skin care. Single-discipline nursing interventions often struggle to comprehensively address these complex pathogenic factors. How to achieve precise prevention and dynamic management of IAD through multidisciplinary collaboration and information technology has become an important research direction in critical care nursing.

The proposal of the predictive nursing concept provides a new approach for IAD prevention and control. This concept emphasizes a shift “from passive response to active prevention” through systematic assessment, risk prediction, and individualized intervention before significant changes occur in the patient’s condition.^[12]^ In recent years, researchers both domestically and internationally have begun to apply the predictive nursing concept to high-risk nursing areas such as pressure ulcers and catheter-associated infections, achieving positive results.^[13]^ However, research on predictive nursing for IAD in ICU patients remains relatively scarce, and integrated models combining multidisciplinary collaboration and informational support have not yet been standardized.

Based on this, this study constructed a “Multidisciplinary Dynamic Nursing Strategy Based on Predictive Nursing” and implemented it in ICU patients. The aim was to explore the impact of this strategy on the incidence, severity, healing status, and quality of nursing care for IAD through measures such as early risk identification, dynamic assessment, multidisciplinary collaboration, and informational intervention, thereby providing a scientific basis and practical reference for protecting skin integrity in ICU patients.

## 2. Research methods

### 2.1. Study design

This study was approved by the Ethics Committee of The First Affiliated Hospital of Hebei North University. This study employed a retrospective cohort design. It retrospectively analyzed nursing records and medical chart data of patients treated in the ICU of a tertiary Grade A hospital to investigate the impact of a multidisciplinary dynamic nursing strategy based on the predictive nursing concept on the incidence and prognostic outcomes of IAD in the ICU. The primary data sources were the nursing information system (NIS) and the electronic medical record (EMR) system. Patients consecutively admitted to the ICU between January 2023 and December 2024 who met the inclusion criteria were selected as the study subjects. Based on the implementation time point (January 2024) of the “Multidisciplinary Dynamic Nursing Strategy Based on Predictive Nursing” rolled out by the hospital’s nursing management department, patients were divided into 2 groups: the control group (n = 132), consisting of patients who received routine care before implementation, and the intervention group (n = 168), consisting of patients who received the multidisciplinary dynamic nursing strategy after implementation.

### 2.2. Inclusion and exclusion criteria

#### 2.2.1. Inclusion criteria

Age ≥18 years, and the first ICU stay duration ≥24 hours; Presence of urinary incontinence, fecal incontinence, or mixed incontinence during hospitalization, indicating a risk of developing IAD; Intact skin upon ICU admission, without primary pressure injuries, abrasions, burns, or other skin damage; Complete clinical data and nursing records, allowing for the tracing of major nursing measures and IAD occurrence; Meeting standard ICU nursing assessment and intervention criteria, and receiving standardized incontinence care management.

#### 2.2.2. Exclusion criteria

Presence of IAD or other severe skin lesions in other areas before ICU admission; Skin damage primarily caused by non-incontinence factors such as pressure injuries, infection, allergy, or drug reactions; Comorbid severe immune system diseases, advanced malignant tumors, or an expected survival period of <48 hours.

## 3. Nursing intervention methods

### 3.1. Control group (routine care)

Patients in the control group were managed according to the routine care model in place at the hospital prior to the implementation of the “Multidisciplinary Dynamic Nursing Strategy Based on Predictive Nursing.” Nursing measures followed the hospital’s existing prevention and management protocol for IAD.^[14]^ These primarily included: inspecting the patient’s skin for moisture and pressure every 2 hours and maintaining detailed records; cleaning soiled areas due to incontinence using pH-neutral, mild cleansers, avoiding products containing irritating or alkaline ingredients; applying zinc oxide ointment or a skin barrier film to the local skin to form a protective barrier and reduce chemical irritation from urine and feces; and appropriately using absorbent pads based on the severity of incontinence to maintain local cleanliness and dryness. Standardized operating procedures were strictly followed throughout the nursing process. New nursing techniques, dressings, or assessment tools were not introduced to maintain the consistency and stability of care measures, thereby reducing the impact of confounding factors on the study results and ensuring the comparability and scientific validity of the control group data.

### 3.2. Intervention group (predictive nursing + multidisciplinary dynamic nursing strategy)

Patients in the intervention group received the Multidisciplinary Dynamic Nursing Strategy based on the predictive nursing concept, in addition to the routine care. This strategy focused on early identification, high-risk warning, dynamic intervention, and multidisciplinary collaboration, utilizing information technology to achieve visualization and continuous optimization of the nursing process. The specific measures were as follows.

#### 3.2.1. Predictive nursing and dynamic risk assessment

The intervention group initiated IAD risk assessment within 24 hours of patient admission to the ICU, using the Perineal Assessment Tool (PAT) for daily scoring, and dynamically adjusted nursing measures based on the risk level. Early warning thresholds were set in the EMR system; when the PAT score was ≥8, the system automatically triggered an enhanced intervention process. The nursing team updated individualized care plans in real time according to risk changes, achieving early identification and continuous intervention for high-risk patients, truly reflecting the proactive and forward-looking nature of predictive nursing.

#### 3.2.2. Multidisciplinary collaborative intervention^[15,16]^

The intervention group established a nurse-led multidisciplinary collaboration mechanism, involving the nursing team, Nutrition Department, Pharmacy Department, Dermatology Department (wound ostomy therapists), and Infection Department. The nursing team was responsible for daily skin cleaning, dressing changes, and skin assessment; the Nutrition Department assessed the patient’s nutritional status and adjusted enteral nutrition plans to reduce the risk of diarrhea and incontinence; the Pharmacy Department optimized antibiotic use strategies to prevent intestinal flora imbalance; the Dermatology and Infection Departments participated in consultations and treatment for patients with severe IAD, guiding local anti-infection measures and dressing selection, thus forming a systematic and collaborative nursing management model (see Supplementary Table 1, Supplemental Digital Content 1).

#### 3.2.3. Individualized nursing measures

The intervention group developed individualized nursing plans based on the patient’s risk level and skin condition. For low-risk patients, the focus was on strengthening skin barrier protection, such as using silicone foam dressings to reduce friction; for moderate- to high-risk patients, new protective dressings (e.g., hydrophilic fiber dressings combined with ostomy powder) were used in combination, and the frequency of repositioning and skin exposure was increased; for patients with infection or skin breakdown, dermatology consultations were promptly requested, and antimicrobial dressings (e.g., silver-ion dressings) were used locally to control infection and promote skin repair.

#### 3.2.4. Informatization nursing support

Relying on the hospital’s NIS and mobile nursing terminals, the intervention group achieved digital management of the nursing process. Nurses could input patients’ daily risk scores and nursing records in real time via mobile terminals, and the system automatically generated and updated dynamic nursing plans. If the PAT score increased for 3 consecutive days, the system automatically sent a multidisciplinary consultation reminder, ensuring timely and precise intervention for high-risk patients. Informatization support not only improved nursing efficiency but also ensured the completeness and traceability of data records.

#### 3.2.5. Implementation of quality control and compliance monitoring

To ensure the effective execution of the intervention measures, the research team conducted unified training for all nurses in the intervention group before strategy implementation. The training content included the predictive nursing concept, use of the PAT tool, standardized operation of skin protection products, and multidisciplinary collaboration processes. Nurses were required to pass theoretical and practical assessments before working independently. During the study period, 10% of patient cases were randomly audited weekly to verify the consistency between nursing records and actual operations, and the information system was used to monitor the timeliness of assessments (e.g., assessments delayed by more than 2 hours were considered noncompliant). Additionally, the research team established a continuous quality improvement mechanism, regularly feeding back compliance data and optimizing nursing processes. All nursing-related data, including IAD incidence, severity, healing time, and nursing costs, were uniformly managed through an electronic database (e.g., REDCap) to ensure authenticity, completeness, and traceability.

### 3.3. Data collection

The data required for this study were primarily sourced from the hospital’s NIS and EMR system. The data collection included the following aspects:

#### 3.3.1. General data

General demographic and clinical data of the patients were collected, including gender, age, weight, body mass index, Acute Physiology and Chronic Health Evaluation II (APACHE II) score, Sequential Organ Failure Assessment score, primary diagnosis upon admission, ICU length of stay, mechanical ventilation usage, type of incontinence (urinary, fecal, or mixed), serum albumin level, use of sedatives and vasoactive drugs, and usage of urinary catheters and fecal management devices.

#### 3.3.2. IAD occurrence

Data on the number of IAD cases, incidence rate, and time of first occurrence during the ICU stay were collected from nursing records, risk assessment forms, and skin care records. If a patient presented with skin moisture, erythema, erosion, etc during hospitalization, the responsible nurse confirmed and recorded it based on IAD diagnostic criteria.

#### 3.3.3. IAD Severity grading^[17]^

The internationally accepted severity grading standard for IAD was used to assess confirmed cases, categorized as mild (localized erythema with intact skin), moderate (localized erosion or superficial breakdown), or severe (significant erosion or exudation, accompanied by pain or secondary infection). All gradings were independently assessed by 2 uniformly trained nurses. In case of disagreement, a wound ostomy therapist reviewed and confirmed the assessment to ensure objectivity and consistency.

#### 3.3.4. IAD healing and prognostic indicators

Healing outcomes of IAD patients were recorded, including the number of completely healed cases, healing time (in days), incidence of nursing-related infections, and ICU length of stay. Healing was defined as the return of skin color, temperature, and integrity to normal, with no symptoms such as exudation, erythema, erosion, or pain. Skin lesions not recurring for more than 48 consecutive hours were considered completely healed. Nursing-related infections referred to secondary infections associated with impaired skin barrier or improper local care, recorded after confirmation by a clinician or the infection department. All healing and infection information was observed and recorded daily by the responsible nurse and reviewed by a wound ostomy therapist.

#### 3.3.5. Nursing compliance and quality control indicators

Data on various nursing performance metrics were extracted from the NIS, including daily risk assessment compliance rate, timely warning response rate (≤2 hours), multidisciplinary consultation trigger rate, nursing record completeness rate, nursing operation consistency pass rate, and data entry timeliness rate. Daily risk assessment compliance rate referred to the proportion of nurses who completed the daily IAD risk assessment as required; timely warning response rate referred to the proportion of interventions completed within 2 hours after a system-triggered risk warning; multidisciplinary consultation trigger rate referred to the proportion of eligible cases that actually underwent consultation; nursing record completeness rate referred to the proportion of nursing documents fully completed without missing items; nursing operation consistency pass rate referred to the proportion of operations consistent with standard protocols; data entry timeliness rate referred to the proportion of data entries completed within 30 minutes after assessment or operation. The system automatically calculated the completion rates for each indicator, and the nursing quality control team regularly conducted random checks to ensure data accuracy and traceability.

#### 3.3.6. Data sorting and verification

All data were entered and cross-checked by 2 researchers to ensure completeness and consistency; missing items were traced or excluded. The data were encrypted and imported into statistical analysis software for processing.

### 3.4. Statistical analysis

All data were analyzed using SPSS 26.0 statistical software. Measurement data, after normality testing, were expressed as mean ± standard deviation (± s), and intergroup comparisons were performed using an independent samples *t* test; data not conforming to normal distribution were expressed as median and interquartile range (M [P25, P75]), and nonparametric tests were used. Count data were expressed as number and percentage [n (%)], and intergroup comparisons were performed using χ^2^ test. All tests were 2-sided, and a *P* < .05 was considered statistically significant.

## 4. Results

### 4.1. Comparison of general data

A total of 300 ICU patients were included, with 132 in the control group and 168 in the intervention group. There were no statistically significant differences between the 2 groups in terms of age (65.3 ± 12.4 years vs 66.1 ± 11.8 years), gender (male 62.1% vs 64.3%), Acute Physiology and Chronic Health Evaluation II score (20.7 ± 5.2 vs 21.1 ± 5.0), BMI, Sequential Organ Failure Assessment score, ICU length of stay, proportion of mechanical ventilation, type of incontinence, albumin level, use of sedatives and vasoactive drugs, or use of urinary and fecal management devices (all *P* > .05), indicating comparability (Table 1).

**Table 1:** Comparison of general characteristics between the 2 groups.

Variable	Control group (n = 132)	Intervention group (n = 168)	Statistic	*P* value
Age (yr)	65.3 ± 12.4	66.1 ± 11.8	*t* = 0.52	.603
Sex (male/female)	82 (62.1)/50 (37.9)	108 (64.3)/60 (35.7)	*χ*^2^ = 0.14	.709
BMI (kg/m^2^)	22.8 ± 3.4	23.0 ± 3.6	*t* = 0.49	.624
APACHE II score	20.7 ± 5.2	21.1 ± 5.0	*t* = 0.64	.526
SOFA score	8.5 ± 2.9	8.7 ± 3.0	*t* = 0.56	.576
Length of ICU stay (d)	14.6 ± 5.7	14.2 ± 5.9	*t* = 0.57	.57
Mechanical ventilation (n [%])	94 (71.2)	124 (73.8)	*χ*^2^ = 0.24	.626
Type of incontinence (n [%])				
Urinary incontinence	48 (36.4)	55 (32.7)	*χ*^2^ = 0.71	.701
Fecal incontinence	34 (25.8)	45 (26.8)		
Mixed incontinence	50 (37.8)	68 (40.5)		
Serum albumin (g/L)	33.2 ± 5.4	32.9 ± 5.1	*t* = 0.49	.626
Sedative use (n [%])	76 (57.6)	101 (60.1)	*χ*^2^ = 0.18	.671
Vasopressor use (n [%])	64 (48.5)	86 (51.2)	*χ*^2^ = 0.23	.633
Urinary catheter placement (n [%])	88 (66.7)	108 (64.3)	*χ*^2^ = 0.16	.69
Fecal management device use (n [%])	52 (39.4)	70 (41.7)	*χ*^2^ = 0.13	.716

APACHE II = Acute Physiology and Chronic Health Evaluation II, BMI = Body Mass Index, SOFA = Sequential Organ Failure Assessment.

### 4.2. Comparison of IAD occurrence

The difference in the incidence of IAD during the ICU stay between the 2 groups was statistically significant. The control group had 38 cases (28.8%) of IAD, while the intervention group had 24 cases (14.3%), indicating a significantly lower incidence in the intervention group (*χ*^2^ = 8.45, *P* = .004) (Table 2). There was no statistically significant difference in the time to IAD occurrence between the 2 groups (6 [4–9] days vs 8 [5–11)] days, *P* = .067).

**Table 2:** Comparison of incontinence-associated dermatitis (IAD) incidence between the 2 groups.

Indicator	Control group (n = 132)	Intervention group (n = 168)	χ^2^ value	*P* value
IAD cases	38 (28.8)	24 (14.3)	8.45	.004
IAD incidence rate (%)	28.8	14.3		
Onset time of IAD (M [P25, P75], d)	6 (4–9)	8 (5–11)	1.83	.067

D = Incontinence-associated dermatitis.

### 4.3. Comparison of IAD severity grading

The distribution of IAD severity between the 2 groups showed a statistically significant difference (*χ*^2^ = 7.62, *P* = .022) (Table 3). In the control group, the majority of cases were mild (22 cases, 57.9%), followed by moderate (11 cases, 28.9%) and severe (5 cases, 13.2%). In contrast, the intervention group was predominantly composed of mild cases (18 cases, 75.0%), with moderate cases numbering 5 (20.8%) and severe cases only 1 (4.2%). The results indicate that the severity of IAD was significantly milder in the intervention group.

**Table 3:** Comparison of IAD severity classification between the 2 groups.

IAD severity	Control group (n = 38)	Intervention group (n = 24)	χ^2^ value	*P* value
Mild	22 (57.9)	18 (75.0)	7.62	.022
Moderate	11 (28.9)	5 (20.8)		
Severe	5 (13.2)	1 (4.2)		

IAD = Incontinence-associated dermatitis.

### 4.4. Comparison of IAD healing time and prognosis

The healing outcomes of IAD in the intervention group were superior to those in the control group. The number of completely healed IAD cases in the intervention group was 22 (91.7%), significantly higher than the 25 cases (65.8%) in the control group (*χ*^2^ = 5.83, *P* = .016). The average healing time in the intervention group was (7.2 ± 2.9) days, significantly shorter than the (9.7 ± 3.6) days in the control group (*t* = 4.08, *P* < .001) (Table 4). There was no statistically significant difference in the incidence of nursing-related infections between the 2 groups (15.8% vs 8.3%, *P* = .377). The ICU length of stay in the intervention group was shorter ([13.1 ± 4.9] days vs [15.4 ± 5.8] days, *t* = 2.04, *P* = .043), and the difference was statistically significant.

**Table 4:** Comparison of IAD healing outcomes and prognosis between the 2 groups.

Indicator	Control group (n = 38)	Intervention group (n = 24)	Statistic	*P* value
Complete healing cases (n [%])	25 (65.8)	22 (91.7)	χ^2^ = 5.83	.016
Healing rate (%)	65.8	91.7		
Healing time (days, x̄±s)	9.7 ± 3.6	7.2 ± 2.9	*t* = 4.08	<.001
Nursing-related infection (n [%])	6 (15.8)	2 (8.3)	*χ*^2^ = 0.78	.377
Length of ICU stay (d, x̄±s)	15.4 ± 5.8	13.1 ± 4.9	*t* = 2.04	.043

SICU = Intensive Care Unit.

### 4.5. Comparison of nursing compliance and quality indicators

The nursing compliance and quality control indicators in the intervention group were significantly better than those in the control group (*P* < .05) (Table 5). Specifically, the daily risk assessment compliance rate in the intervention group was 94.6%, significantly higher than the 71.2% in the control group. The timely warning response rates were 91.7% and 64.4%, respectively. The multidisciplinary consultation trigger rate was 57.1% in the intervention group, compared to 36.4% in the control group. Additionally, the nursing record completeness rate (97.0% vs 89.4%), nursing operation consistency pass rate (94.0% vs 83.3%), and data entry timeliness rate (93.5% vs 76.5%) were all significantly improved in the intervention group (*P* < .01). These results indicate that the predictive multidisciplinary dynamic nursing strategy effectively enhances nursing compliance and quality.

**Table 5:** Comparison of nursing compliance and quality indicators between the 2 groups.

Indicator	Control group (n = 132)	Intervention group (n = 168)	*χ*^2^ value	*P* value
Daily risk assessment compliance	94 (71.2)	159 (94.6)	33.12	<.001
Timely early-warning response (≤2 h)	85 (64.4)	154 (91.7)	34.78	<.001
Multidisciplinary consultation triggered	48 (36.4)	96 (57.1)	11.24	.001
Completeness of nursing documentation	118 (89.4)	163 (97.0)	6.55	.01
Consistency of nursing operations	110 (83.3)	158 (94.0)	8.91	.003
Timely data entry rate	101 (76.5)	157 (93.5)	15.69	<.001

## 5. Discussion

This study, through a retrospective cohort design, investigated the impact of a multidisciplinary dynamic nursing strategy based on the predictive nursing concept on the incidence and prognostic outcomes of IAD in ICU patients. The results demonstrated that this strategy holds significant advantages in reducing IAD incidence, alleviating severity, shortening healing time, and improving nursing compliance and quality. This indicates that the dynamic intervention model combining predictive nursing and multidisciplinary collaboration can effectively optimize skin management for incontinent ICU patients and improve their clinical outcomes.

The results of this study showed that the IAD incidence in the intervention group was 14.3%, significantly lower than the 28.8% in the control group (*P* = .004). Multicenter studies have indicated that implementing risk assessment-based proactive nursing interventions can reduce IAD incidence by approximately 40%.^[18]^ Domestic studies have also confirmed^[19]^ that applying predictive nursing processes significantly reduces the risk of skin damage in incontinent ICU patients. The core of predictive nursing lies in “early identification and early prevention,” utilizing dynamic risk assessment tools (such as the PAT score) to promptly identify high-risk patients and implement graded interventions, thereby achieving a shift from passive care to active management. This study set risk warning thresholds within the NIS and reinforced implementation through electronic reminders, further enhancing the timeliness and continuity of interventions.^[20,21]^ The results showed that the daily risk assessment compliance rate and timely warning response rate in the intervention group were significantly higher than those in the control group (*P* < .001), indicating that informationalized predictive nursing has clear advantages in improving the efficiency of risk identification.^[22]^ Multidisciplinary collaboration is an important means for solving complex nursing problems. The intervention group in this study established a dynamic collaboration mechanism involving nursing, nutrition, pharmacy, dermatology, and infection control. The study results showed that the severity of IAD in the intervention group was significantly milder than that in the control group (*P* = .022), with the proportion of severe IAD being only 4.2%, much lower than the 13.2% in the control group. This aligns with the internationally proposed “multidimensional skin integrity management model,” which emphasizes comprehensive intervention from multiple aspects such as skin barrier, moisture management, nutritional support, and microbial control, significantly improving IAD prevention and treatment outcomes.^[23–26]^ Furthermore, the participation of the Nutrition and Pharmacy Departments helped reduce high-risk factors like diarrhea and intestinal flora imbalance at the source. Consultations from the Dermatology and Infection Departments optimized local dressing and anti-infection treatment plans for critically ill patients. Multidisciplinary collaboration not only enhanced the scientific and systematic nature of interventions but also strengthened the comprehensive judgment ability of the nursing team, enabling patients to receive more precise and individualized nursing support.

In this study, the intervention group achieved visualization and dynamic management of the nursing process with the support of the information system. All risk assessments, intervention records, and consultation triggers were traceable. The results showed that the nursing record completeness rate (97.0% vs 89.4%), operation consistency (94.0% vs 83.3%), and data entry timeliness rate (93.5% vs 76.5%) in the intervention group were significantly better than those in the control group (*P* < .01). This indicates that informational support plays a positive role in improving nursing compliance and quality control. Furthermore, the automatic statistical and quality control mechanisms of the information system can form a continuous quality improvement closed loop, enabling continuous optimization of the nursing strategy, which aligns with the trend of refined management in modern critical care nursing.^[27,28]^

This study found that the complete healing rate of IAD patients in the intervention group was significantly higher than that in the control group (91.7% vs 65.8%, *P* = .016), and the average healing time was significantly shorter (7.2 ± 2.9 days vs 9.7 ± 3.6 days, *P* < .001). This is consistent with another study reporting that proactive moisture control and barrier repair strategies can accelerate skin healing.^[29]^ Predictive nursing, through early identification and precise intervention, helps reduce the duration of skin exposure to urine and fecal stimuli, thereby promoting skin barrier repair and reducing the risk of secondary infection.^[30]^ Additionally, the ICU length of stay in the intervention group was approximately 2.3 days shorter than that in the control group (*P* = .043), suggesting that this nursing model not only improved local skin condition but may also have indirectly enhanced overall recovery efficiency by reducing complications, which is of significant importance for the rational utilization of critical care resources. The predictive nursing concept emphasizes a “risk-oriented” approach and data-driven decision-making, aligning with the development direction of evidence-based nursing and precision medicine. The multidisciplinary dynamic nursing strategy in this study fully embodies the closed loop management model of “active identification - real-time intervention - continuous improvement.” It not only effectively reduced the incidence and severity of IAD but also enhanced nursing quality and team collaboration efficiency.^[29,30]^ This model can provide a replicable and scalable paradigm for skin management in high-risk environments such as the ICU.

This study is a single-center retrospective study with a limited sample size and still carries some risk of bias. Although confounding factors were reduced through strict inclusion/exclusion criteria and quality control processes, it might still be influenced by subjective factors such as the completeness of nursing records and implementation compliance. Future multicenter, prospective randomized controlled studies could be conducted to further validate the universality and long-term effects of the predictive nursing-based multidisciplinary dynamic intervention in different types of ICU populations. Furthermore, integrating artificial intelligence risk prediction models and wearable monitoring technology may further enhance the accuracy of IAD risk warning and the intelligence level of nursing interventions.

## 6. Conclusion

The results of this study indicate that the multidisciplinary dynamic nursing strategy based on the predictive nursing concept can significantly reduce the incidence of IAD in ICU patients, alleviate its severity, promote healing, and improve nursing compliance and quality. By integrating information tools and multidisciplinary resources, this model provides a scientific, systematic, and sustainable nursing pathway for skin management in critically ill patients, demonstrating high clinical promotion value.

## Author contributions

**Conceptualization:** Shuwei Huang, Xuebin Hao, Yang An, Yimiao Wang, Jiantong Liu.

**Data curation:** Shuwei Huang, Xuebin Hao, Yang An, Yimiao Wang.

**Formal analysis:** Shuwei Huang, Xuebin Hao, Yang An, Yimiao Wang.

**Funding acquisition:** Shuwei Huang, Yimiao Wang.

**Investigation:** Shuwei Huang.

**Writing—original draft:** Shuwei Huang, Yang An, Yimiao Wang, Jiantong Liu.

**Writing—review & editing:** Shuwei Huang, Yang An, Yimiao Wang, Jiantong Liu.


