# Supplementary material for: Research on multidisciplinary dynamic nursing strategies for incontinence-associated dermatitis in ICU patients based on predictive nursing
Source: Medicine (Baltimore). 2026 Aug 14;105(33):e48619. doi: 10.1097/MD.0000000000048619 (PMC13480902; doi:10.1097/MD.0000000000048619)
Supplement: Supplementary file 1 [file medi-105-e48619-s001.docx]

Supplemental Table 1. Multidisciplinary Team Responsibilities and Scope of Work

| **Professional Team** | **Core Responsibilities** | **Specific Scope of Work** |
| --- | --- | --- |
| ICU Nursing Team | Risk Assessment, Measure Implementation, Information Coordination | 1. Complete the initial PAT assessment within 24 hours of admission, followed by daily assessments and documentation. |
|  |  | 2. Initiate enhanced preventive measures and label the patient as high-risk when the PAT score is ≥8. |
|  |  | 3. Execute standardized skin care: use pH-neutral cleansers, gently pat dry, and properly apply skin protectants or prophylactic dressings. |
|  |  | 4. Responsible for communicating changes in patient skin condition and risk to other disciplines and implementing the comprehensive care plan. |
| Clinical Nutrition Department | Nutritional Assessment & Support, Diarrhea Risk Reduction | 1. Assess patient nutritional status (e.g., using NRS-2002) and serum albumin levels. |
|  |  | 2. For patients with diarrhea or at high risk, review and adjust enteral nutrition regimens, including switching to formulas containing dietary fiber or short peptides, and optimizing infusion rates. |
|  |  | 3. Monitor patient bowel tolerance to reduce the occurrence of incontinence at the source. |
| Clinical Pharmacy Department | Medication Regimen Optimization, Drug-Associated Diarrhea Risk Reduction | 1. Review medications that may cause diarrhea or gut microbiota dysbiosis, such as broad-spectrum antibiotics and laxatives. |
|  |  | 2. Provide recommendations for optimizing antimicrobial therapy (e.g., de-escalation therapy) based on susceptibility results and patient condition. |
|  |  | 3. Assist in adjusting other medications that may affect bowel function. |
| Dermatology / Wound Ostomy Care Nurses | Complex Wound Management & Technical Guidance | 1. Consult on moderate-to-severe IAD cases presenting with skin breakdown, significant exudate, or failure to respond to routine care. |
|  |  | 2. Provide professional wound assessment and management plans. |
|  |  | 3. Guide the selection and proper application of advanced functional dressings (e.g., hydrocolloid fiber dressings, silver-containing dressings). |
| Hospital Infection Management Department | Infection Surveillance & Prevention | 1. Guide the collection of wound secretion samples for microbiological culture when signs of infection appear in IAD wounds. |
|  |  | 2. Guide appropriate topical or systemic anti-infective therapy based on culture results and drug resistance data. |
|  |  | 3. Supervise the implementation of infection control measures, such as contact isolation. |
